# Brain Functional and Structural Changes in Alzheimer's Disease With Sleep Disorders: A Systematic Review

**DOI:** 10.3389/fpsyt.2021.772068

**Published:** 2021-11-01

**Authors:** Yong-shou Liu, Yong-ming Wang, Ding-jun Zha

**Affiliations:** ^1^Department of Otolaryngology-Head and Neck Surgery, Xijing Hospital, Air Force Medical University, Xi'an, China; ^2^Department of Psychosis Studies, Institute of Psychiatry, Psychology & Neuroscience, King's College London, London, United Kingdom

**Keywords:** Alzheimer's, sleep disorders, brain function and structure, default mode network, hippocampus

## Abstract

**Introduction:** Sleep disorders (SLD) are supposed to be associated with increased risk and development of Alzheimer's disease (AD), and patients with AD are more likely to show SLD. However, neurobiological performance of patients with both AD and SLD in previous studies is inconsistent, and identifying specific patterns of the brain functional network and structural characteristics in this kind of comorbidity is warranted for understanding how AD and SLD symptoms interact with each other as well as finding effective clinical intervention. Thus, the aims of this systematic review were to summarize the relevant findings and their limitations and provide future research directions.

**Methods:** A systematic search on brain functional and structural changes in patients with both AD and SLD was conducted from PubMed, Web of Science, and EMBASE databases.

**Results:** Nine original articles published between 2009 and 2021 were included with a total of 328 patients with comorbid AD and SLD, 367 patients with only AD, and 294 healthy controls. One single-photon emission computed tomography study and one multislice spiral computed tomography perfusion imaging study investigated changes of cerebral blood flow; four structural magnetic resonance imaging (MRI) studies investigated brain structural changes, two of them used whole brain analysis, and another two used regions of interest; two resting-state functional MRI studies investigated brain functional changes, and one 2-deoxy-2-(18F)fluoro-d-glucose positron emission tomography (18F-FDG-PET) investigated 18F-FDG-PET uptake in patients with comorbid AD and SLD. Findings were inconsistent, ranging from default mode network to sensorimotor cortex, hippocampus, brain stem, and pineal gland, which may be due to different imaging techniques, measurements of sleep disorder and subtypes of AD and SLD.

**Conclusions:** Our review provides a systematic summary and promising implication of specific neuroimaging dysfunction underlying co-occurrence of AD and SLD. However, limited and inconsistent findings still restrict its neurobiological explanation. Further studies should use unified standards and comprehensive brain indices to investigate the pathophysiological basis of interaction between AD and SLD symptoms in the development of the disease spectrums.

## Introduction

Alzheimer's disease (AD) is a severe neurodegenerative disease, manifested as deterioration in cognition (1), memory (2), thinking, behavior, and the ability to perform everyday activities (3). The World Health Organization reports that, as the most common form of dementia, AD may contribute to 30–35 million patients worldwide and 6–7 million new cases every year. However, there is still no particularly effective treatment for it (3). Finding possible risk or comorbid factors and effective intervention of this disease is warranted.

As one of the most important noxious factors on physical and mental health (4), sleep disorder (SLD) is reported to be closely associated with development of AD (5–7). Some researchers even believe that SLD has decisive effects on AD (2). It is reported that roughly more than 50% of patients with AD have got significant SLD (8), which may include insomnia, obstructive apnea hypopnea syndrome (OSA), rapid eye movement sleep behavior disorder (RBD), sleep apnea, shortened sleep duration, fragmented sleep, slow wave sleep disruption, and misalignment of circadian rhythm (9). Older adults with SLD are more likely to have a diagnosis of AD or cognitive decline (10). Brain chemical changes in OSA patients could further lead to cognitive impairment and early AD clinical performance, and continuous positive airway pressure therapy could modify these AD biomarkers (11). RBD may also lead to cognitive impairment and pathologies of AD (12).

Another 9-year follow-up study found reduced sleep was associated with a two-fold increased risk of AD compared with controls (13). The reasons for this significant correlation could be that the SLD can increase stress reactions and impair attention, episodic memory (14) and cognitive function (15), increase adrenocorticotropin and cortisol secretion, weaken neuronal structures, increase risk of cell death (5). Specifically, SLD could increase beta-amyloid production and deposition (2) and decrease its clearance in the brain (7), further cause synaptic dysfunction (2), and exacerbate the deterioration of AD symptoms, especially when both were severe (9). One review indicates that the treatment of SLD may also alleviate cognitive decline and the risk of AD (2) and be an effective therapy for AD symptoms (9). However, the specific neurobiological basis of the interactive process between AD and SLD symptoms are still controversial.

Neuroimaging technology could help us understand the brain pathophysiology of AD and SLD *in vivo*. Previous review and meta-analysis studies indicate that patients with AD exhibit decreased regional cerebral blood flow in the posterior cingulate cortices (PCC), precuneus, inferior parietal lobules (IPL), dorsolateral prefrontal cortex (DLPFC) (16); decreased gray matter volume in the left parahippocampal gyrus, left PCC, right fusiform gyrus, and right superior frontal gyrus (SFG) (17) and network changes mainly associated with subcortical areas (18) and default mode network (DMN) (19). In particular, the DMN is reported as the first network affected by AD (20). Meanwhile, patients with SLD show decreased brain functional and structural indices in the frontal cortex, temporal gyrus, fusiform gyrus, striatum, cingulate cortex, precentral gyrus, and reduced glucose metabolism in the limbic system as well as increased brain activation and regional homogeneity in the middle frontal gyrus, precunus, cingulate gyrus (21) and overactivation in the hypothalamic-pituitary-adrenal cascade (5). However, one review indicates that cortical hyperarousal other than hypo-arousal activities, as the central feature of SLD, may be located at the temporal cortex and hippocampus and could contribute to both AD and SLD pathogenesis so as to increase their comorbidity rate (8).

Although these findings show some shared and bidirectional interactions of brain disturbances, neurodegeneration, and neuropathology between AD and SLD (9), the key brain changes associated with high comorbidity and their interactions are still controversial and remain to be elucidated. The necessity and merits of this review are that through the conclusions of previous brain functional and structural findings in patients with comorbid AD and SLD, we could find the possibly abnormal SLD-related brain regions or networks that beta-amyloid mainly deposit or neural inflammation led to in these patients. We could also well-understand the specific neurobiological basis of how SLD contributes to the development of AD symptoms and find a possible effective way to delay or prevent the brain alterations and clinical manifestations of AD through effective treatment of SLD symptoms. Furthermore, concluding the specific differences of brain changes in patients with comorbidity of AD and SLD compared with patients with AD alone could also help us find a possible specific subtype of the AD spectrum. For these aims, the present review provides an overview of the specific neuroimaging findings of patients with comorbid AD and SLD, their limitations, and future research directions. In addition, if the number of included studies using the same imaging technologies is >3, then a meta-analysis will be conducted.

Previous studies mainly found abnormal limbic structures, especially hippocampal astrophic (9) and dysfunctional regions in the DMN in patients with AD or SLD (20). Based on these findings, we hypothesize that these brain regions and networks would exhibit relatively consistent abnormality in patients with comorbid AD and SLD in related studies.

## Methods

### Search Strategy and Study Selection

A systematic literature search was conducted to identify published studies that examined brain imaging changes in patients with AD and SLD using databases of PubMed, Web of Science, and Embase. The key words were searched using the following terms: (sleep OR insomnia OR apnea hypopnea) AND (Alzheimer's^*^) AND (MRI OR brain imag^*^), and the search period ended on August 8, 2021, from the earliest date of the databases. The reference lists of retrieved articles were also searched for identifying potentially relevant studies. There were no other restrictions, such as SLD classification, imaging methods, language, or publication year; i.e., we included all studies of insomnia, OSA, RBD, sleep apnea, shortened sleep duration, fragmented sleep, slow wave sleep disruption, and misalignment of circadian rhythm, etc. All primary studies that reported brain functional or structural changes of the patients with comorbid AD and SLD were included in the review. If values of demographic information or brain changes were unclear, we attempted to contact the authors for clarification. The exclusion criteria were (1) case reports, (2) review or meta-analyses, and (3) non-human studies, and (4) if studies had overlapping samples, only studies with the largest sample were included. Any divergences were resolved by discussion between authors.

### Data Extraction

The extracted characteristics of the articles included first author, publish year, sample size, mean age of participants, questionnaires for AD or SLD in the articles, imaging technique, whole brain or regions of interest analysis, country of study, and the main results, which were brain functional or structural or chemical changes in comorbid AD and SLD compared with AD or healthy controls.

## Results

### Included Studies

**Figure 1:**
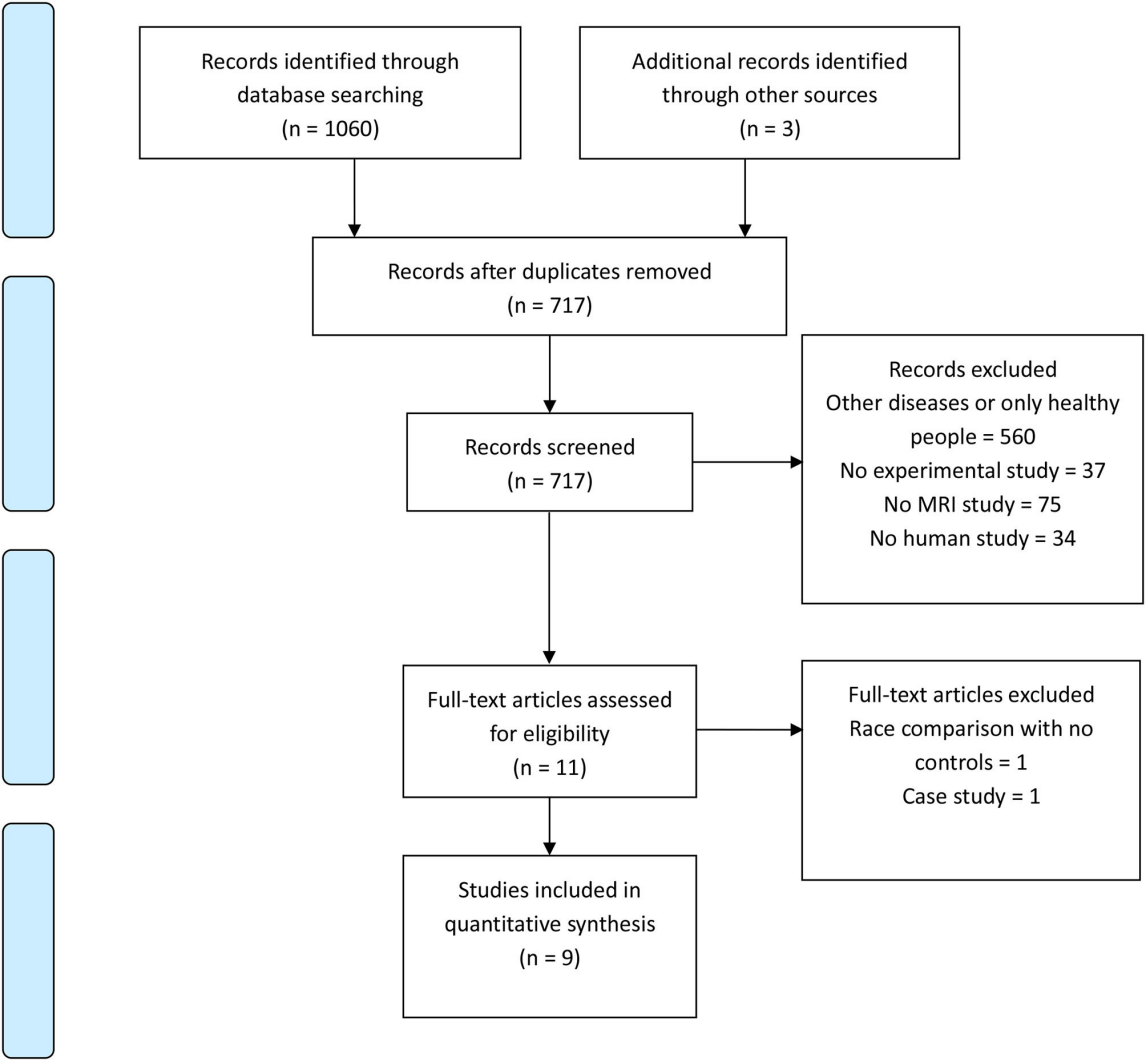
PRISMA Flow diagram of studies selection for review.

A PRISMA flow chart of article choice is presented in Figure 1. A total of 1,240 articles were retrieved, 1,231 articles were excluded, and nine studies were finally included in the review. Summary of the included studies is showed in Table 1.

**Table 1:** Summary of AD&SLD studies included in the meta-analysis.

**References**	**Country**	**AD and SLD subsets**	**AD and SLD cases (male/female)**	**AD cases (male/female)**	**Controls (male/female)**	**AD and SLD mean age (SD)**	**AD mean age (SD)**	**Controls mean age (SD)**	**Questi-onnaire**	**Imaging technique**	**ROIs**	**Main results**
Ismail et al. (22)	Canada	AD with or without sleep loss	14/24	7/10	17/20	72.4 (7.4)	74.2 (8.3)	71.5 (6.3)	NINCD, sNPI	SPECT-CBF	WB	Higher perfusion in the right MFG in AD and SLD compared with AD
Lee et al. (24)	Korea	sNPI in AD & SLD	13/31	N.A.	14/26	76.2 (5.7)	N.A.	75.2 (4.7)	NINCD, CDR, sNPI	3T T1-weighted sMRI	Brain stem	Decreased volume in the brain stem; negative correlation between the sNPI scores and left posterior lateral brain stem volume in AD and SLD
Liguori et al. (25)	Italy	Polysomnographic recordings in AD & SLD	8/18	N.A.	7/11	71.56 (3.92)	N.A.	74.11 (2.78)	NIAAA, PSG	18F-FDG-PET	Hypo-thalamus	Interplay between the reduction of sleep efficiency, REM sleep and the reduction of hypothalamic 18F-FDG-PET uptake, which negatively correlated with the index of neurodegeneration in AD and SLD
Yi et al. (27)	Korea	Poor or good sleeper AD	32	14	N.A.	N.A.	N.A.	N.A.	PSQI	3T T1-weighted sMRI	WB and hippo subfields	Decreased gray matter volume in the bilateral cingulate gyrus in poor sleeper AD. No hippo findings.
Matsuoka et al. (28)	Japan	AD with or without sleep disturbance	8/11	11/33	N.A.	81.5 (6.4)	77.8 (6.9)	N.A.	NINCD, ICD-10, sNPI	3T T1-weighted sMRI	WB and pineal gland	Decreased gray matter volume in the bilateral precuneus in AD with sleep disturbance. No pineal gland findings.
Li et al. (23)	China	AD with poor or normal sleep	7/9	9/5	53/70	74.66 (8.50)	70.76 (8.24)	74.02 (7.13)	NINCD, CDR, NPI-Q	rs-fMRI &PET	WB	Increased dALFF variance in the right cerebellum, left STP, right rectus, right hippo, decreased dALFF variance in the right SMG, decreased sALFF in the left precentral gyrus, right IPL and cuneus in AD and SLD compared with AD
Park et al. (20)	Korea	AD with or without sleep behavior disorder	8/31	79/178	N.A.	76.8 (7.4)	77.4 (7.4)	N.A.	CERAD, RBDSQ	3T T1-weighted sMRI & PET	Pineal gland	Decreased pineal gland volume in AD & SLD compared with AD, and the pineal gland volume negatively correlated with the severity of SLD
Liu et al. (26)	China	AD with sleep disorders	31/45	N.A.	32/44	73.07 (5.41)	N.A.	72.32 (5.28)	CCMD-3, PQSI	MSCTP-CBF	TL, FL, hippo, BG	Decreased CBF in the TL, FL, hippo and BG in AD and SLD
Wang et al. (29)	China	Mild AD with or without sleep disturbances	12/26	8/13	N.A.	73.7 (7.2)	73.6 (8.4)	N.A.	NIAA, CDR, PSQI	rs-fMRI	WB	Decreased mPerAF and PerAF in the left brainstem, left calcarine gyrus, left lingual gyrus; decreased PerAF in the left fusiform gyrus, left parahippocampal gyrus, left precentral gyrus, left postcentral gyrus

*18F-FDG-PET, 2-deoxy-2-(18F)fluoro-d-glucose positron emission tomography; AD, Alzheimer's disease; BG, basal ganglia; CBF, cerebral blood flow; CCMD-3, Chinese Classification of Mental Disorders version 3; CDR, clinical dementia rating scale; CERAD, consortium to establish a registry for Alzheimer's disease assessment packet clinical assessment battery; dALFF, dynamic amplitude of low-frequency fluctuation; FL, frontal lobe; hippo, hippocampus; ICD-10, International Classification of Disease version 10; IPL, inferior parietal lobule; MFG, middle frontal gyrus; mPerAF, percent amplitude of fluctuation divided by global mean percent amplitude of fluctuation; MSCTP, multi-slice spiral computed tomography perfusion imaging; N.A., not available; NIAA, National Institute of Aging-Alzheimer's criteria in 2011; NIAAA, National Institute on Aging-Alzheimer's Association workgroups on diagnostic guidelines for Alzheimer's disease; NINCD, National Institute of Neurological and Communicative Disorders and stroke – Alzheimer's disease and related disorders association criteria; NPI-Q, brief questionnaire form of neuropsychiatric inventory; PerAF, percent amplitude of fluctuation; PSQI, Pittsburgh sleep quality index; RBDSQ, rapid eye movement sleep behavior disorder screening questionnaire; PSG, polysomnographic recordings; REM, rapid eye movement; ROI, region of interests; rs-fMRI, resting state functional magnetic resonance imaging; sALFF, static amplitude of low-frequency fluctuation; SD, standard deviation; SLD, sleep disorders; SMG, supramarginal gyrus; sMRI, structural magnetic resonance imaging; sNPI, sleep subscale of neuropsychiatric inventory; SPECT, single-photon emission computed tomography; STP, superior temporal pole; TL, temporal lobe; WB, whole brain*.

Two of them compared brain differences between the comorbidity of AD and SLD groups, the AD group and healthy controls (22, 23); three studies only included the comorbidity group and healthy controls (24–26); four studies recruited the comorbidity group and the AD group (20, 27–29). Published years of articles ranged from 2009 to 2021. Three studies were conducted in China, three in Korea, one in Canada, one in Italy, and one in Japan.

Included patients with comorbid AD and SLD, AD only, and controls in this review were 328, 367, and 294, respectively. Sample size of every group ranged from 14 to 257. Mean age of participants ranged from 71.5 to 81.5.

In these studies, one single-photon emission computed tomography (SPECT) (22) study and one multislice spiral computed tomography perfusion imaging (MSCTP) (26) study investigated changes of cerebral blood flow; two whole-brain structural magnetic resonance imaging (sMRI) studies investigated brain structural changes (27, 28), one sMRI study investigated the brain stem volume (24), and another investigated the pineal gland volume (30); two resting-state functional magnetic resonance imaging (rs-fMRI) studies investigated brain functional changes (23, 29); and one 2-deoxy-2-(18F) fluoro-d-glucose positron emission tomography (18F-FDG-PET) investigated 18F-FDG-PET uptake in patients with comorbid AD and SLD (25).

### Evaluation of AD and SLD

Diagnosis and evaluation of AD and SLD are heterogeneous. In AD diagnosis, four studies used National Institute of Neurological and Communicative Disorders and stroke—AD and related disorders association criteria (NINCD) (22–24, 28), one used National Institute on Aging-Alzheimer's Association workgroups on diagnostic guidelines for AD (NIAAA) (25), one used a consortium to establish a registry for AD assessment packet clinical assessment battery (CERAD) (30), one used Chinese Classification of Mental Disorders version 3 (CCMD-3) (26), one used National Institute of Aging-Alzheimer's criteria in 2011 (NIAA) (29), and one did not provide a diagnostic standard (27). In SLD evaluation, three used a sleep subscale of neuropsychiatric inventory (sNPI) (22, 24, 28), three used the Pittsburgh sleep quality index (PSQI) (26, 27, 29), one used polysomnographic recordings (PSG) (25), one used a brief questionnaire form of neuropsychiatric inventory (NPI-Q) (23), one used a rapid eye movement sleep behavior disorder screening questionnaire (RBDSQ) (30).

### Brain Changes in Comorbid AD and SLD

All studies found significant brain changes in patients with comorbid AD and SLD compared with patients with only AD or healthy controls.

One region of interest (ROI)-based sMRI study found significant differences of posterior brain stem morphology in the AD and SLD group compared with controls and a negative correlation between the NPI scores and left posterior lateral brain stem volume (24), and another ROI study found decreased pineal gland volume in AD and SLD compared with AD, and the pineal gland volume negatively correlated with the severity of SLD (30). One whole brain and hippocampus subfield ROI study found decreased gray matter volume of bilateral cingulate gyrus in poor sleeper AD compared with AD only as well as no significant results in the hippocampus (27). Another whole brain and pineal gland ROI study found decreased gray matter volume of bilateral precuneus in AD patients with sleep disturbance compared with patients with AD only, which negatively correlated with the sNPI scores (28). However, different from the second study, no significant results were found in the pineal gland ROI comparison (28).

Two rs-fMRI studies investigated brain functional changes (23, 29). One of them found increased dynamic amplitude of low-frequency fluctuation (dALFF) variance of right cerebellum, left superior temporal pole (STP), right rectus, right hippocampus, decreased dALFF variance of right supramarginal gyrus (SMG), decreased sALFF of left precentral gyrus, right IPL, and cuneus in patients with AD and SLD compared with AD, and dALFF variance positively correlated with amyloid deposit in regions involved in memory and sleep (23). Another study found a decreased percentage of amplitude of fluctuation (PerAF) and PerAF divided by global mean PerAF (mPerAF) in the left brainstem, left calcarine gyrus, left lingual gyrus; decreased PerAF in the left fusiform gyrus, left parahippocampal gyrus, left precentral gyrus, left postcentral gyrus in patients with AD and SLD compared with AD (29).

In the rest of the studies, one MSCTP study investigated changes of cerebral blood flow (CBF) in ROIs, and found decreased CBF of temporal lobe, frontal lobe, hippocampus, and basal ganglia in the AD and SLD group compared with controls (26). One SPECT study found higher CBF in the right middle frontal gyrus (MFG) in the AD and SLD group compared with the AD group (22). One 18F-FDG-PET study investigated 18F-FDG-PET uptake in patients with comorbid AD and SLD and found correlation between the reduction of sleep efficiency, REM sleep, and the reduction of hypothalamic 18F-FDG-PET uptake, which negatively correlated with the index of neurodegeneration in AD and SLD group compared with controls (25).

## Discussion

Nine experimental studies, which investigated brain functional and structural changes in patients with both AD and SLD, using rs-fMRI, sMRI, PET, SPECT, or MSCTP scanning, were included in our review. Although many findings showed heterogeneity, which may be due to small sample sizes, different experimental designs, disease assessment and imaging tools, some findings still support our hypothesis.

DMN is a brain network most activated in the resting state and associated with autobiographical and episodic memory (1), consolidating individual experiments, self-referential information processing, and constructing integrated “self” (31). These cognitive domains are believed to be impaired early, and the DMN is the first dysfunctional network affected by increased beta-amyloid deposition in patients with AD (1, 20) as well as in patients with SLD (32, 33). The abnormal DMN, especially the overactivated hippocampus and medial temporal lobe, is reported to associate with dysfunction of emotion (34), memory, and cognition in patients with SLD (15, 35) and could also interact with increased beta-amyloid production and greater local deposition (1) and facilitate the following hypo-activation and atrophy of hippocampus and other DMN regions in patients with AD (8). However, whether overactivation (8, 15), hypo-activation (26, 29), or no change (22, 27) of the hippocampus in the different developmental processes of these two diseases is still controversial. It may be due to different categories of hippocampal subregions, such as previous studies that believe that changed anterior, medial, or posterior parts of the hippocampal volumes could have different changes in patients with AD (36) or abnormal hippocampal subregions of the dentate gyrus (DG), cornu ammonis 1/2/3, and subiculum play distinct roles in memory impairments (37) or due to different neurobiological changes of SLD subtypes, such as OSA or RBD, which need further investigation with high-resolution brain imaging.

Decreased sALFF in the precentral gyrus (23) and decreased PerAF in the precentral and postcentral gyrus were found in the comorbidity of the AD and SLD group (29). These decreased precentral activations may imply increased night awakening, reduced slow-wave activity, and sleep depth linked to sleep disruption (38) as well as impaired sensory and motor function by SLD in the development of AD (29).

Decreased volume (24), mPerAF and PerAF of the brainstem were found in the comorbidity group in previous studies (29). The brainstem is usually unmentioned or not investigated in brain imaging studies. However, it is a key neurobiological basis of sleep regulation, producing and regulating the sleep–wake rhythms (29). Degeneration of it could occur very early in patients with AD and also contribute to the onset of SLD, fragmented sleep, impaired memory consolidation, and cognitive decline (24).

Some researchers believe that the pineal gland is associated with sleep regulation through melatonin synthesis, and beta-amyloid could easily inhibit its function (39), which, in turn, could reduce the protection of cholinergic neurons from amyloid toxicity, increase the risk of both SLD and AD (30). In the included studies, only one found decreased pineal gland volume in the comorbidity group (30). Another ROI-based study (28) and four whole-brain studies did not find any significant results (22, 23, 27, 29). These inconsistent results in previous studies may imply a different presentation of melatonergic systems in different developmental stages or subtypes of the diseases, which need more studies with large sample sizes for further verification (28).

Some other positive results could also be worth considering. One whole-brain study found higher CBF in the dorsal lateral prefrontal cortex (DLPFC) in the comorbidity group compared with the AD group (22). Another ROI-based study found significant decreased brain glucose consumption in the hypothalamus in the comorbidity group, which negatively correlated with the index of neurodegeneration in patients with AD and SLD and a significant interplay between the reduction of sleep efficiency, REM sleep, and the reduction of hypothalamic 18F-FDG-PET uptake (25). The abnormal activation of DLPFC, which may be the result of impaired thalamocortical ascending executive cholinergic pathways, could associated with sleep loss and frequent arousals during sleep (22) and lead to cognitive dysfunction (15). The hypothalamus is associated with homeostasis and the sleep–wake cycle and could be affected by AD pathology leading to some non-cognitive deficits, such as sleep–wake and neuroendocrine disorders and loss of weight (25). Meanwhile, hypothalamic-pituitary-adrenal axis dysregulation and inflammation was also reported to contribute to the development of pathophysiology of both SLD and AD symptoms (8). The knowledge of the hypothalamic circuitry functions in the interaction between AD and SLD symptoms still needs expansion for reliable biomarkers and therapeutic approaches.

There are still many limitations left in current research. First, the clinical manifestation and developmental process of SLD is complicated with high heterogeneity, and the assessment of it is still not unified between studies. Furthermore, many studies did not report the onset time or duration of SLD. Second, neurobiological explanation of comorbidity is still constrained by a lack of magnetic resonance spectroscopy or other microscopic neurobiological studies. Elaborate experiments with solution of these limitations, such as large, well-characterized, or homogeneous samples; standard assessment; diagnosis; imaging and analysis protocols (40); and longitudinal studies on AD risk management, prevention and intervention from the viewpoint of SLD treatment are warranted (6). Finally, because the number of studies included in our review using the same scanning technology are all not greater than three, meta-analysis was not able to be conducted. It limits the preciseness and generalizability of our research.

Integrating all these studies together, we could conclude that SLD may have induced even decisive effects on the pathological development of AD symptoms. The DMN, especially the hippocampus as well as the sensorimotor cortex, may be the initial neurophysiological basis of interactions between AD and SLD symptoms. These changes could be mainly associated with memory, self-awareness, and sensorimotor deficiencies. Functions of the brainstem and pineal gland in this comorbidity still need further verification. Our study may contribute to a better understanding of the pathophysiology and possible risk of AD in patients with SLD and a suggestion of potential neural biomarkers for the further investigation on the early diagnosis and treatment of SLD patients with AD risk or with the comorbidity of AD and SLD. The facilitated effects of SLD symptoms on AD, and this physiological basis in the AD developmental continuum from subclinical to high-risk population and to patients deserve more attention in the future studies. For example, elaborately task-based fMRI experimental design, technology of magnetic resonance spectroscopy or AD animal models could be introduced. Findings from these studies could further help us to prevent or attenuate cognitive decline and AD symptoms (2), perform targeted early intervention on possible dementia risk and improve senile life quality (5).

## Data Availability Statement

The original contributions presented in the study are included in the article/supplementary material, further inquiries can be directed to the corresponding author/s.

## Author Contributions

Y-sL and Y-mW designed the study, managed the literature searches and the analyses, and wrote the whole manuscript. D-jZ designed the study, interpreted the data, and edited this manuscript. All authors have contributed to approved the final manuscript.

## Conflict of Interest

The authors declare that the research was conducted in the absence of any commercial or financial relationships that could be construed as a potential conflict of interest. The handling editor YZ declared a shared affiliation, though no other collaboration, with one of the authors Y-sL and D-jZ at the time of the review.

## Publisher's Note

All claims expressed in this article are solely those of the authors and do not necessarily represent those of their affiliated organizations, or those of the publisher, the editors and the reviewers. Any product that may be evaluated in this article, or claim that may be made by its manufacturer, is not guaranteed or endorsed by the publisher.

## Abbreviations

18F-FDG-PET: 2-deoxy-2-(18F)fluoro-d-glucose positron emission tomography
AD: Alzheimer's disease
CBF: cerebral blood flow
CCMD-3: Chinese Classification of Mental Disorders version 3
CERAD: consortium to establish a registry for Alzheimer's disease assessment packet clinical assessment battery
dALFF: dynamic amplitude of low-frequency fluctuation
DLPFC: dorsolateral prefrontal cortex
DMN: default mode network
IPL: inferior parietal lobules
MFG: middle frontal gyrus
mPerAF: percent amplitude of fluctuation divided by global mean PerAF
MSCTP: multi-slice spiral computed tomography perfusion imaging
NIAA: National Institute of Aging-Alzheimer's criteria in 2011
NIAAA: National Institute on Aging-Alzheimer's Association workgroups on diagnostic guidelines for Alzheimer's disease
NPI-Q: brief questionnaire form of neuropsychiatric inventory
OSA: obstructive apnea hypopnea syndrome
PCC: posterior cingulate cortices
PerAF: percent amplitude of fluctuation
PSG: polysomnographic recordings
PSQI: Pittsburgh sleep quality index
RBD: rapid eye movement sleep behavior disorder
RBDSQ: rapid eye movement sleep behavior disorder screening questionnaire
ROI: region of interest
rs-fMRI: resting-state functional magnetic resonance imaging
SFG: superior frontal gyrus
SLD: sleep disorder
SMG: supramarginal gyrus
sMRI: structural magnetic resonance imaging (sMRI)
sNPI: sleep subscale of neuropsychiatric inventory
SPECT: Single-photon emission computed tomography
STP: superior temporal pole.
